# Formulation of enzyme blends to maximize the hydrolysis of alkaline peroxide pretreated alfalfa hay and barley straw by rumen enzymes and commercial cellulases

**DOI:** 10.1186/1472-6750-14-31

**Published:** 2014-04-26

**Authors:** Ajay Badhan, Yuxi Wang, Robert Gruninger, Donald Patton, Justin Powlowski, Adrian Tsang, Tim McAllister

**Affiliations:** 1 Agriculture and Agri food Canada, Lethbridge research Centre, Lethbridge, Alberta, Canada; 2 Centre for Structural and Functional Genomics, Concordia University, Montreal, Quebec H4B 1R6, Canada

**Keywords:** Glycosyl hydrolases, Esterases, Fungi, Rumen, Biofuel, Straw, Alfalfa

## Abstract

**Background:**

Efficient conversion of lignocellulosic biomass to fermentable sugars requires the synergistic action of multiple enzymes; consequently enzyme mixtures must be properly formulated for effective hydrolysis. The nature of an optimal enzyme blends depends on the type of pretreatment employed as well the characteristics of the substrate. In this study, statistical experimental design was used to develop mixtures of recombinant glycosyl hydrolases from thermophilic and anaerobic fungi that enhanced the digestion of alkaline peroxide treated alfalfa hay and barley straw by mixed rumen enzymes as well as commercial cellulases (Accelerase 1500, A1500; Accelerase XC, AXC).

**Results:**

Combinations of feruloyl and acetyl xylan esterases (FAE1a; AXE16A_ASPNG), endoglucanase GH7 (EGL7A_THITE) and polygalacturonase (PGA28A_ASPNG) with rumen enzymes improved straw digestion. Inclusion of pectinase (PGA28A_ASPNG), endoxylanase (XYN11A_THITE), feruloyl esterase (FAE1a) and β-glucosidase (E-BGLUC) with A1500 or endoglucanase GH7 (EGL7A_THITE) and β-xylosidase (E-BXSRB) with AXC increased glucose release from alfalfa hay. Glucose yield from straw was improved when FAE1a and endoglucanase GH7 (EGL7A_THITE) were added to A1500, while FAE1a and AXE16A_ASPNG enhanced the activity of AXC on straw. Xylose release from alfalfa hay was augmented by supplementing A1500 with E-BGLUC, or AXC with EGL7A_THITE and XYN11A_THITE. Adding arabinofuranosidase (ABF54B_ASPNG) and esterases (AXE16A_ASPNG; AXE16B_ASPNG) to A1500, or FAE1a and AXE16A_ASPNG to AXC enhanced xylose release from barley straw, a response confirmed in a scaled up assay.

**Conclusion:**

The efficacy of commercial enzyme mixtures as well as mixed enzymes from the rumen was improved through formulation with synergetic recombinant enzymes. This approach reliably identified supplemental enzymes that enhanced sugar release from alkaline pretreated alfalfa hay and barley straw.

## Background

Cellulosic sugars are the primary currency of the bio economy. Lignocellulose is the most abundant source of biomass on the planet and represents an enormous storehouse of sugars. Monosaccharides from biopolymers of lignocellulosic feedstock can be fermented into a wide variety of biofuels and biochemicals including ethanol, organic acids (e.g., lactic acid), solvents (e.g., acetone, butanol), 5-hydroxymethyl furfural, levulinic acid and lubricants [1]. Hemicellulose can also be used to produce ethanol, xylite, furfural, nylons and plant gums such as thickeners, adhesives and stabilizers [1]. Canada, with its enormous forests and huge annual amounts of agriculture residues, is a huge producer of cellulosic biomass. The weight of straw produced from cereal crops equals or exceeds that of grain, and represents a significant opportunity as a biorefinery feedstock to contribute to environmental, social and economic sustainability [1]. Similarly, recent work has shown that alfalfa is 2 ~ 3 times more efficient than corn or soybean as a biomass source owing to its high biomass yield, perennial nature, fixation of aerial nitrogen, and production of valuable co-products, making it a model forage species for biofuel research [2]. However, cellulosic material is remarkably recalcitrant; making the release of fermentable sugars with current hydrolysis options commercially cost prohibitive [3].

A crucial step in the bioconversion of lignocellulosic feedstock to biofuels and biomaterials is to maximize the saccharification of cellulose and hemicellulose components to fermentable sugars in a manner that is cost effective. One of the challenges is the high cost of enzymes involved in the saccharification of the lignocellulose and the loss of some of the hemicellulose sugars during pre-treatment. Most acidic and alkaline pretreatments (e.g., dilute acid, steam explosion, ammonia recycle percolation) remove a significant fraction of hemicellulose and/or lignin, thereby enhancing enzyme accessibility. While pretreatments like ammonia fiber expansion (AFEX) do not physically extract hemicellulose or lignin as separate fractions, they do modify the cell wall ultra-structure through mechanisms that are currently not well understood [4]. It is reasonable to assume that varying types of pretreated biomass would require specific mixtures of enzymes that were tailor-made for efficient hydrolysis [5]. Similarly, in order to minimize costs pertaining to enzyme production, identification of major enzymes and optimization of their relative ratios could reduce enzyme usage without sacrificing the rate or yield of substrated during hydrolysis [6]. Likewise, plant cell walls are a primary source of nutritional energy for ruminants, but with many forages less than 50% of the cell wall fraction is digested [7]. Substantial benefits would be realized if a greater percentage of this potential energy was made available for fermentation in the rumen through an increase in the digestibility of the plant cell wall fraction.

This study was designed to examine the ability of key fungal enzymes from *Aspergillis niger* and *Thielavia terrestris,* in combination with commercial enzymes (Accellerase 1500 and Accellerase XC) or mixed rumen enzymes to further enhance the breakdown of alkaline peroxide (AP) pretreated alfalfa hay and barley straw. AP pretreatment was selected as it accomplishes a degree of delignification, with relatively low environmental impact and without the need for special reaction chambers [8]. The process causes selective removal of lignin and xylan through a combination of lignin oxidation and de-acetylation and also decreases cellulose crystallinity, enhancing the susceptibility of plant cell walls to enzymatic degradation [9]. However, conservation of acetyl and feruloyl ester linkages and only partial lignin oxidation has been reported at the low concentrations (≤2.0%) used in this study [10]. Therefore, we utilized a selection of purified auxiliary enzymes (i.e., esterase (AXE16A_ASPNG, AXE16B_ASPNG, FAE 1a); pectinase (PGA28A_ASPNG); α-arabinofuranosidase (ABF54B_ASPNG); endoglucanase GH7 (EGL7A_THITE); endoxylanase (XYN11A_THITE); β-glucosidase (E-BGLUC) and β-xylosidase (E-BXSRB) to explore their ability to enhance the activity of commercial enzyme and rumen enzyme mixtures. A similar approach using a combination of statistical design, robotic dispensing of substrate slurry and high throughput micro plate techniques to assess enzymatic hydrolysis at comparable protein to biomass loads and reaction volumes has been reported earlier [11]. The high throughput micro assay adopted in this study is based on Chundawat et al. [11], a procedure which has been standardized for solid delivery in biomass slurries, mass transfer related parameters, reproducibility and validity through comparision to conventional National Renewable Energy Laboratory protocols [12].

## Results and discussion

### Formulation of enzyme mixtures for effective hydrolysis of alkaline peroxide treated alfalfa hay

#### Glucose release from enzymatic hydrolysis of AP alfalfa

**Figure 1:**
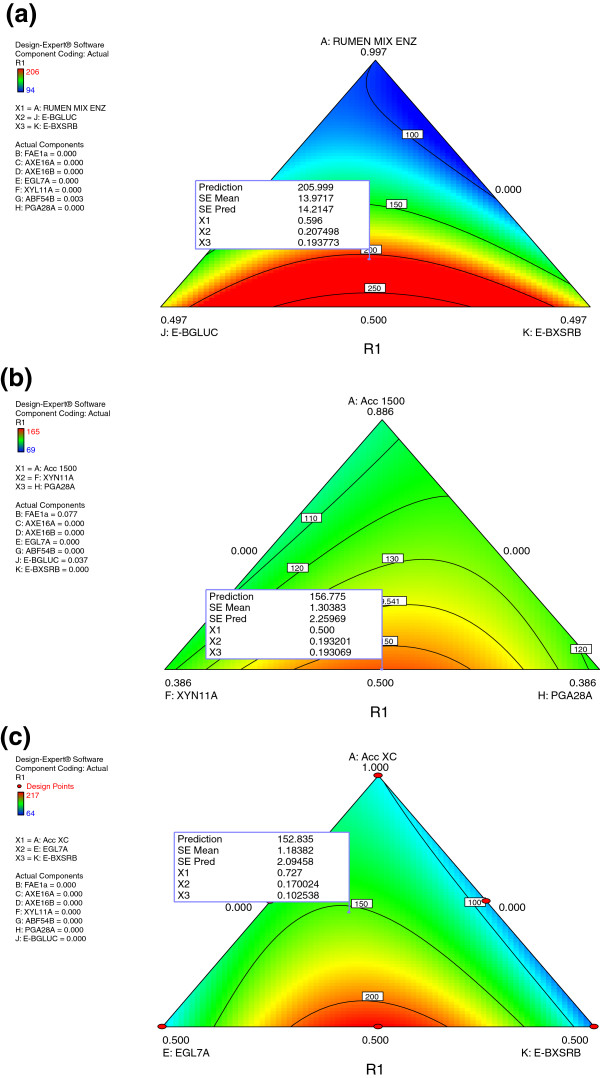
**Ternary plot of model developed to predict relative glucose yield as a function of synergetic interaction of mixed rumen enzymes (a), Accellerase 1500 (b), Accellerase XC (c) with recombinant fungal enzymes for hydrolysis of alkaline peroxide pretreated alfalfa.** Micro assays were performed with loads of 15 mg/g of enzyme and 0.5% biomass.

Compared to rumen enzymes alone, a combination of rumen enzymes (60%) with β-glucosidase (E-BGLUC; 20%) and β-xylosidase (E-BXSRB; 20%) resulted in a two fold increase (Figure 1a and Additional file 1a) in glucose release from AP alfalfa hay. These results suggest a positive synergetic interaction between rumen enzymes with β-glucosidase and β-xylosidase. The observed increase in glucose yield with supplementary β-glucosidase (E-BGLUC) and β-xylosidase (E-BXSRB) activity reflects possible feedback inhibition of rumen cellulase by oligomers released as results of enzymatic digestion. It has been well documented that the presence of sufficient β-glucosidase is important in reducing the inhibition of cellulose by cellobiose [13,14]. The reason for suboptimal β glucosidase activity in the rumen enzyme preparation is unknown, but may reflect the fact that the enzyme mixture used originated from the fluid fraction of rumen contents. It is well known that significant quantities of beta-xylosidase and beta-glucosidase activity are associated with the particulate fraction of rumen contents. It is also possible that in the rumen, intact bacteria uptake cellobiose directly from rumen fluid thereby preventing it from accumulating to levels that impede cellulose digestion [15].

The model predicted optimized enzyme mixtures containing Accellerase 1500 (50%) with polygalacturonase (PGA28_ASPNG: 20%), endo-xylanase (XYN11A_THITE; 20%), feruloyl esterase (FAE 1a; 7%) and β-glucosidase (E-GLUC; 3%) resulting in higher glucose release as compared to control (100% Accellerase 1500) (Figure 1b and Additional file 1b). These results agree with a recent report identifying hemicellulose as an important physical barrier to enzymatic hydrolysis of cellulose, as it blocks enzyme access to the cellulose surface [16]. Hemicellulose, particularly in the form of xylan and its oligomers, has been documented to inhibit cellulase activity even more than the accumulation of glucose or cellobiose [16]. The present study indicates that more efficient cellulose hydrolysis would be achieved by formulating Accellerase XC (73%) with endoglucanase (EGL7A_THITE: 17%) and beta-xylosidase (E-BXSRB; 10%) activity (Figure 1c and Additional file 1c) as compared to Accellerase XC alone. Enhanced glucose release as a result of the addition of EGL7A may be attributed to additional cellulase activity or to the broad substrate specificity exhibited by members of GH7. These endoglucanases have been reported to readily act on xylan, arabinoxylan [17] and xyloglucan [18]. The phylogenetic analysis among the catalytic cores of GH7 enzymes in the CAZy database suggests that GH7 endoglucanases and xylanase may have diverged from a common ancestral gene, possible resulting in GH7 endoglucanases retaining remnants of xylanse activity [18]. These results can be explained by hypothesis that GH7 improves overall cell wall conversion through exposing more xylan trapped within the cell wall matrix, increasing the release of xylo-oligomers. It has been reported that xylo-oligomers are strong inhibitors of cellulase and inclusion of β-xylosidase (*Trichoderma*) reduces the inhibitory impact of these end products on cellulose hydrolysis [16]. Furthermore, xylanases (*Thermoascus aurantiacus*) alone have been shown to enhance the bioconversion of both cellulose and hemicellulose [19].

#### Xylose release from AP pre-treated alfalfa

**Figure 2:**
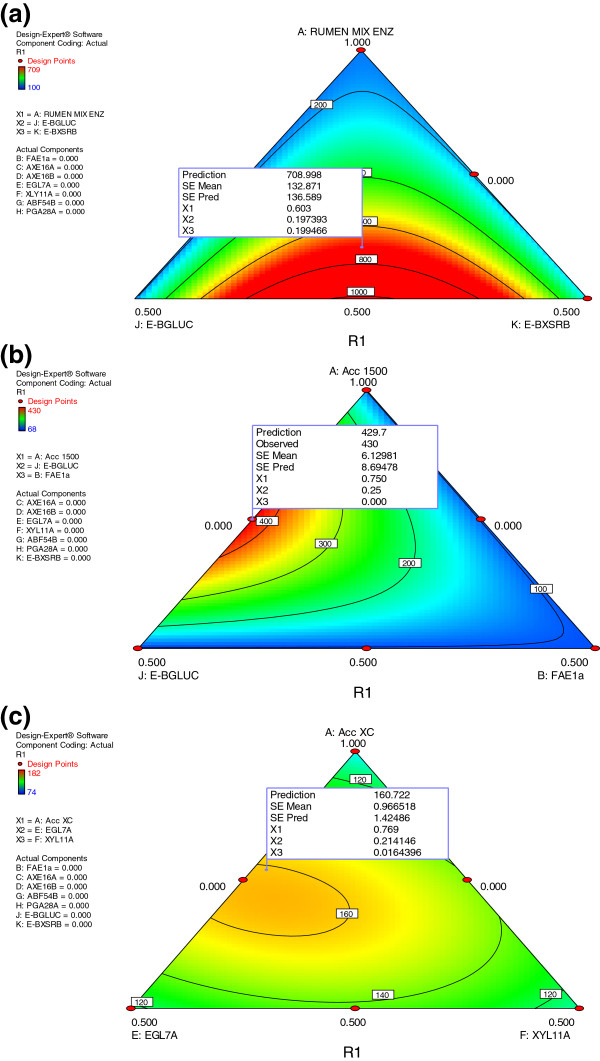
**Ternary plot of model developed to predict relative xylose yield as a function of synergetic interaction of rumen enzymes mix (a), Accellerase 1500 (b), Accellerase XC (c) with recombinant fungal enzymes for hydrolysis of alkaline peroxide pre-treated alfalfa.** Micro assays were performed with loads of 15 mg/g of enzyme and 0.5% biomass.

A seven fold increase in xylose release from alfalfa (Figure 2a and Additional file 2a) was predicted when mixed rumen enzymes (60%) were complemented with β-glucosidase (E-GLUC; 20%) and β-xylosidase (E-BXSRB; 20%). Furthermore, the model predicted a four-fold increase in xylose release (Figure 2b and Additional file 2b) when Accellerase 1500 (75%) was complemented with β-glucosidase (E-GLUC; 25%). Interestingly, optimum xylose yield did not require the addition of xylanase, suggesting that there was sufficient xylanase already in Accellerase 1500. Of the commercial enzyme mixtures investigated, Accellerase XC contained the highest xylanase activity (2500–3800 acid birchwood xylanase units (ABXU/g), and consequently was expected to be most efficient at xylan hydrolysis. However, complementing Accellerase XC with endoglucanase (EGL7A_THITE; 21%) resulted in 1.6 fold higher xylose yield (Figure 2c and Additional file 2c) from AP pretreated alfalfa as compare to Accellerase XC alone. These results suggest improved xylan hydrolysis with increased cellulose solubility and reflect the layered structural complexity of cellulose and xylan in the plant cell wall.

### Formulation of enzyme mixtures for effective hydrolysis of alkaline peroxide treated barley straw

#### Glucose release from AP pretreated barley straw

**Figure 3:**
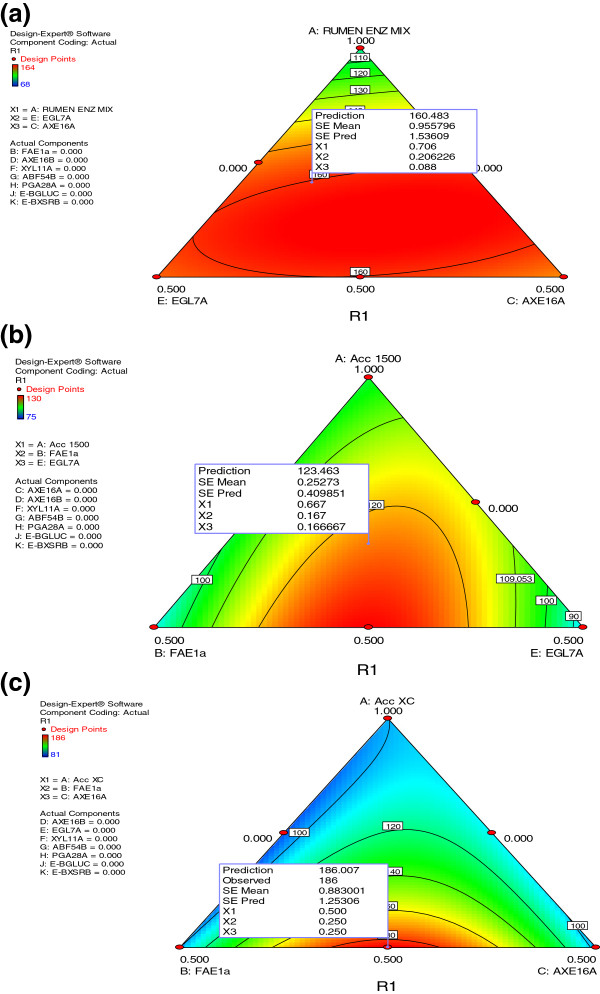
**Ternary plot of model developed to predict relative glucose yield as a function of synergetic interaction with mixed rumen enzymes (a), Accellerase 1500 (b), Accellerase XC (c) with recombinant fungal enzymes for hydrolysis of alkaline peroxide pre-treated barley straw.** Micro assays were performed with 15 mg/g of enzyme and 0.5% biomass.

Maximum glucose yield (1.6 fold higher than control) from barley straw was observed (Figure 3a and Additional file 3a) when mixed rumen enzymes (70%) were combined with family 7 endoglucanase (EGL7A_THITE; 21%) and acetyl xylan esterase (AXE16A_ASPNG; 9%). Glucose yield from Accellerase 1500 was nominally (1.2 fold) higher when it was mixed with feruloyl esterase (FAE1a; 17%) and endoglucanase (EGL7A_THITE; 17%) (Figure 3b and Additional file 3b). Approximately 1.86 fold more glucose was released (Figure 3c and Additional file 3c) by supplementing Accellerase XC (50%) with feruloyl esterase (FAE1a; 25%) and acetyl esterase (AXE16A_ASPNG; 25%). These results demonstrate the importance of esterase activity for holocellulose hydrolysis in barley straw. Our results also resonate with an earlier report [20,21] of conservation of acetyl and feruloyl ester linkages in hot water pretreated corn stover. It has also been documented that peroxide treatment at low concentrations (≤2.0%; as used in this study) did not result in notable lignin oxidation, whereas treatment with H_2_O_2_ at ≥3.0% resulted in significant cleavage of carboxyl groups [10]. Our results are consistent with the notion that ferulic acid linkages to lignin and /or other xylan/pectin chains are in part responsible for recalcitrance of cellulose microfibrils even after mild oxidative treatment. Cleavage of these linkages seems to be imperative in providing access of both cellulases and xylanases to the core of the cell wall matrix [10,20].

#### Xylose release from pre-treated barley straw

**Figure 4:**
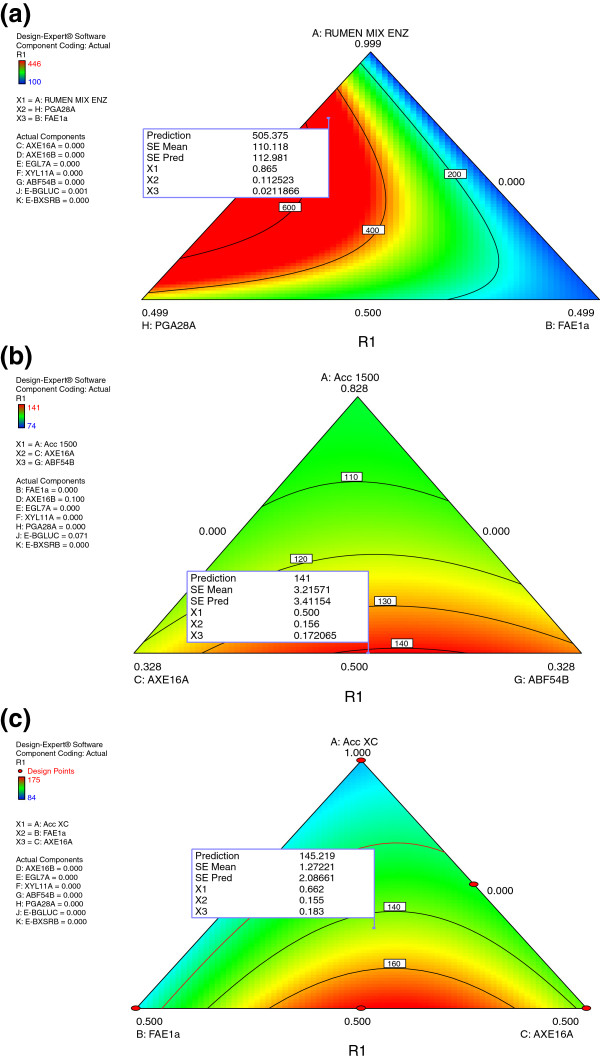
**Ternary plot of model developed to predict relative xylose yield as a function of synergetic interaction of mixed rumen enzymes (a), Accellerase 1500 (b), Accellerase XC (c) with recombinant fungal enzymes for hydrolysis of alkaline peroxide pre-treated barley straw.** Micro assays were performed with 15 mg/g of enzyme and 0.5% biomass.

Xylose release from barley straw was five-fold higher when mixed rumen enzymes (87%) were complemented with polygalacturonase (PGA28A_ASPNG; 11%) and feruloyl esterase (FAE1a; 2%) (Figure 4a and Additional file 4a). This suggests that cross-linked pectin/xylan limited the digestion of barley straw. Furthermore, an optimized mixture containing Accellerase 1500 (50%), arabinofuranosidase (ABF54B_ASPNG; 17%), 16% and 10% acetyl-xylan-esterase AXE16A_ASPNG and AXE16B_ASPNG respectively, and 7% E-BGLUC, was predicted to release 1.4 fold more xylose (Figure 4b and Additional file 4b) from barley straw than Accellerase 1500 alone. Likewise complementing Accellerase XC (66%) with feruloyl esterase (FAE1a; 16%) and acetyl-xylan-esterase (AXE16A_ASPNG; 18%) resulted in the highest xylose yield (Figure 4c and Additional file 4c) from barley straw as compared to Accellerase XC alone. These results suggest that cross-linked polymers with lignin are the primary recalcitrant structure inhibiting the conversion of barley straw to fermentable sugars. Synergistic interaction between feruloyl esterase and xylanase to increase the release of ferulic acid as well as synergetic interaction between feruloyl esterase, xylanase, cellulase, arabinofuranosidase and arabinanases to increase the release of reducing sugar has been reported by others [22].

Our results are consistent with previously reported correlations between lignin phenotype (lignin content and ferulate crosslinking) and enzymatic deconstruction of monocot (barley) and dicot (alfalfa) cell walls. With dicots (alfalfa), a strong negative correlation has been shown between initial lignin content and cellulolytic decomposition of dilute acid pretreated fibrous substrates [23]. Our model also suggests that efficient deconstruction of delignified AP alfalfa can be achieved by core enzymes alone, while feedback inhibition by released products was also an important factor controlling sugar release. With barley as a monocot, digestibility has been reported to not depend on initial lignin content, but rather the degree of ferulate bridges was strongly negatively correlated to digestibility [19,24]. This observation was reflected by our results where esterase activity was shown to be critical for the comprehensive enzymatic deconstruction of barley straw.

**Figure 5:**
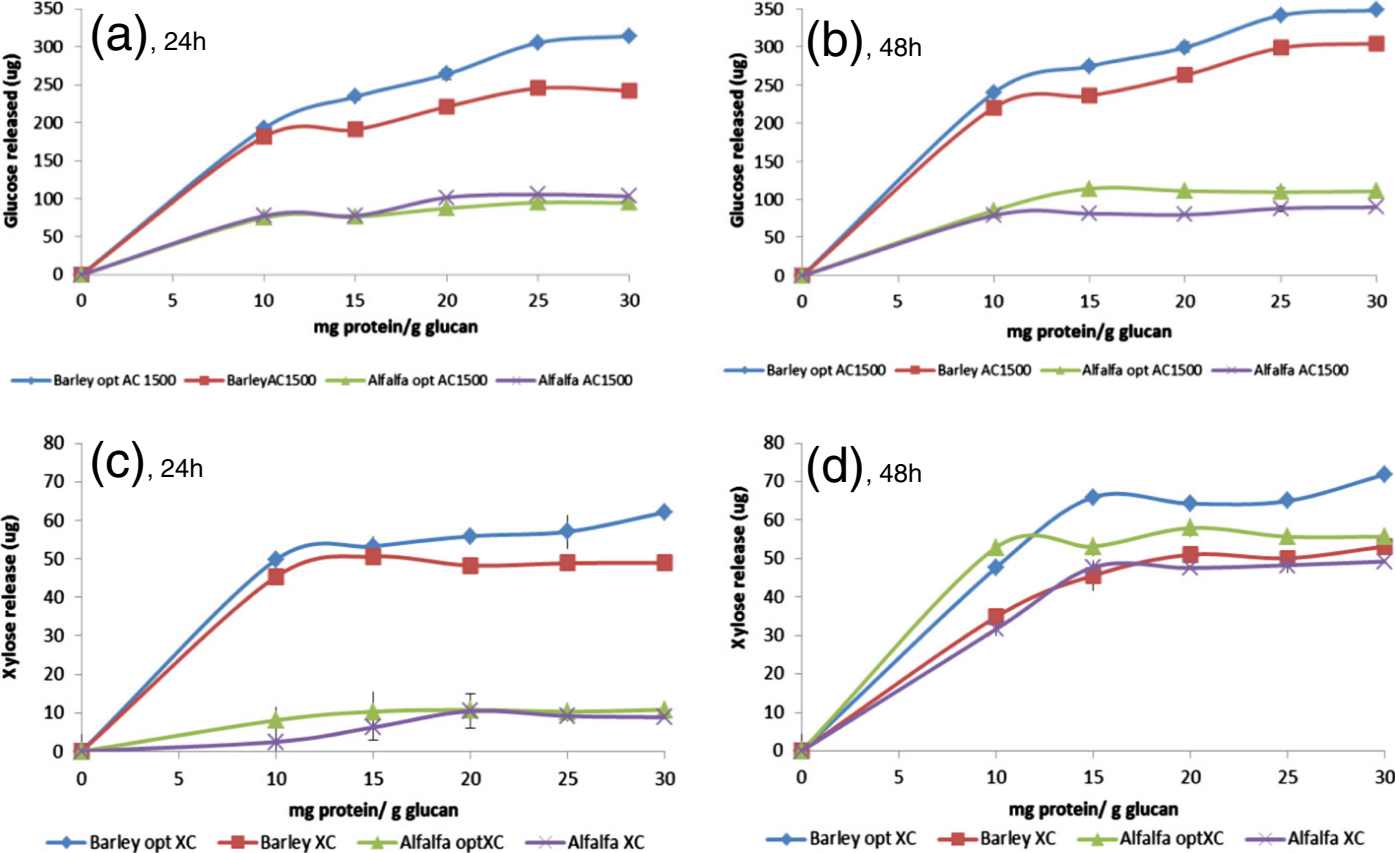
**Glucose and xylose released from treated barley straw and alfalfa (biomass load: 0.5%) as function of fungal enzyme loading at 24 h (a, c) and 48 h (b, d) of incubation.** Error bars (often invisible) represent SD of the mean (n = 8).

**Figure 6:**
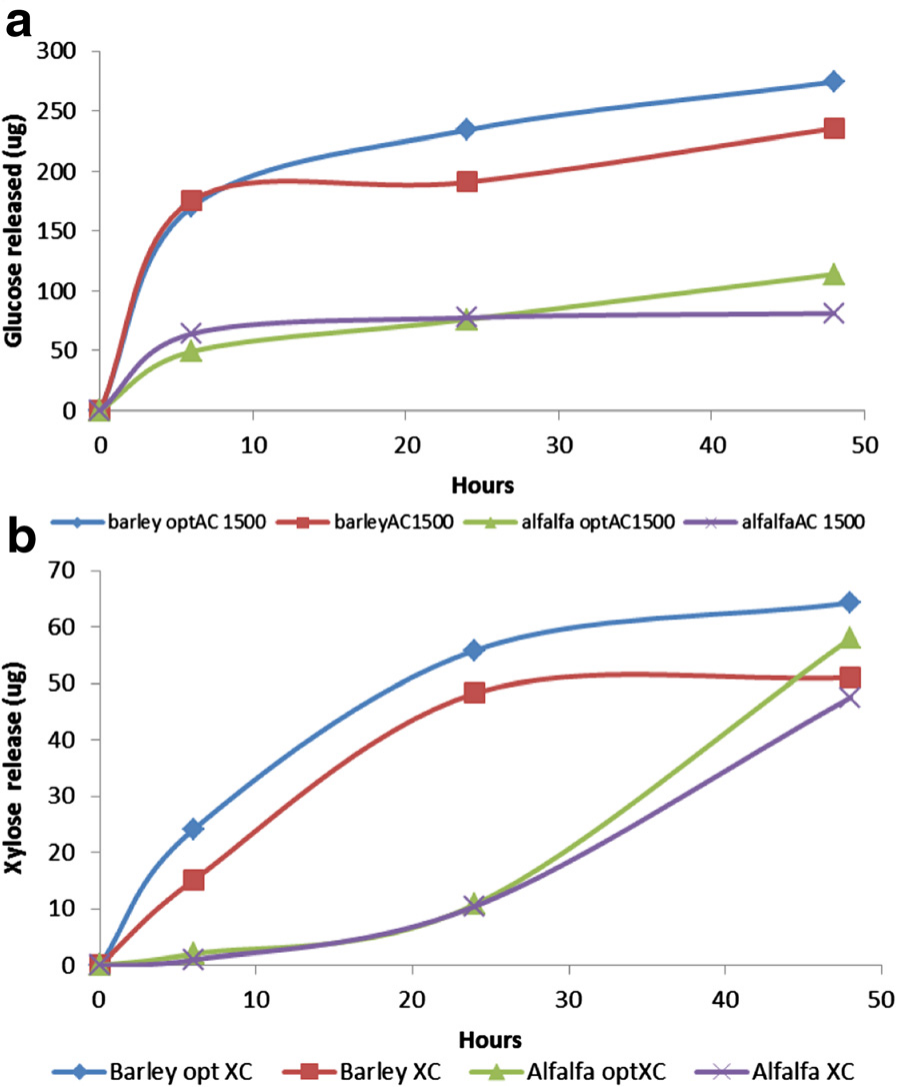
**Time course of glucose (a) and xylose (b) released from treated barley and alfalfa.** All enzymes were included at 15 mg/g glucan with 0.5% biomass.

To validate predictions made by statistical design, the profile of glucose and xylose released from AP pretreated barley and alfalfa as a function of incubation time and protein concentration were further studied. At 6 h, glucose release from AP treated barley as well as alfalfa was observed to be nearly linear for all four mixtures with increasing enzyme concentration (data not shown). For AP treated barley straw, optimized Accellerase 1500 mix was superior (p < 005) to Accellerase 1500 at 24 h (Figure 5a). Although both optimized Accellerase 1500 and Accellerase 1500 showed similar yields after 24 h, and 48 h of incubation with alfalfa hay, the optimized Accellerase 1500 mixture resulted in higher (p < 005) glucose yield from AP treated alfalfa (Figure 5b). At 48 h, with all four enzyme mixtures, sugar release tended to plateau at protein loads above 20 mg/g glucan, with optimized Accellerase 1500 exhibiting the highest glucose release from AP treated barley straw (Figure 5b). Similarly optimized Accellerase XC for barley straw and alfalfa hay, were also observed to enhance xylose release (p < 005) from both substrates (Figure 5c, d). Plotting data collected at 15 mg/g of glucan against time showed that optimized enzyme mixtures released more glucose and xylose than the original commercial enzyme preparations at terminal incubation (Figure 6a and b).

**Figure 7:**
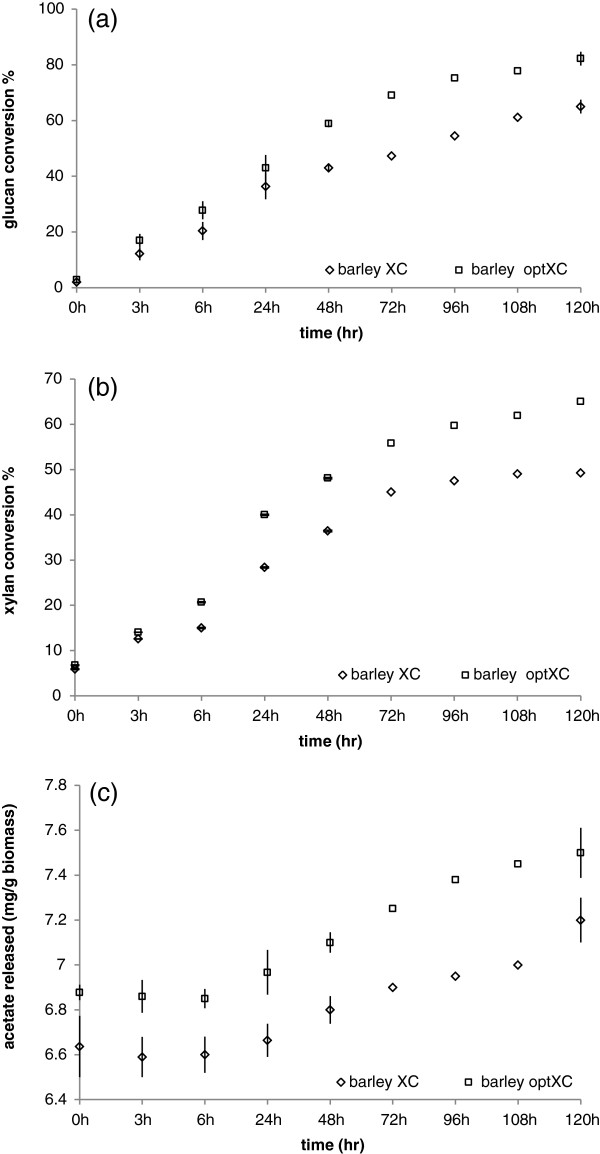
**Glucose conversion (a), xylose conversion (b) and acetate released (c) from pretreated barley at 2% biomass load over 120 h of incubation with 15 mg/g of enzyme.** Error bars represent SD of the mean (n = 8).

**Figure 8:**
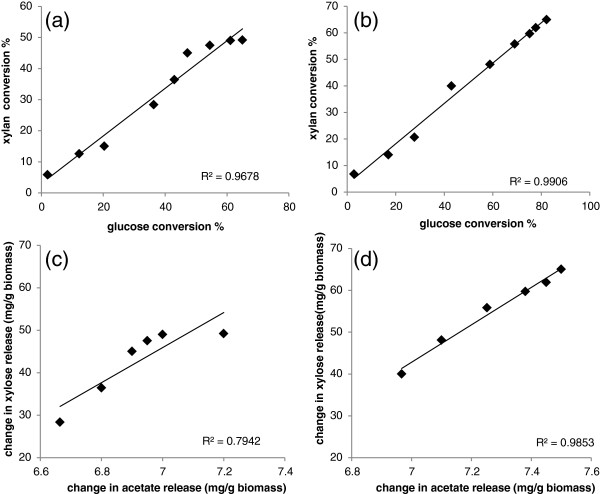
**Change in glucan conversion plotted against change in xylan conversion for Accellerase XC (a) and optimized Accellerase XC (b) over 120 h of incubation with pretreated barley straw (2% biomass load).** Xylose released plotted against acetate released by Accellerase XC **(c)** and optimized Accellerase XC **(d)**, from pretreated barley straw (2% biomass load) over 120 h of incubation. Optimized Accellerase XC composition was identical to those used in Figure 3c. Enzymes were included at 15 mg/g glucan. Error bars represent SD of the mean (n = 8).

Modeled predictions were further validated with a scale up assay using (2% w/v) AP treated barley straw in a 2 ml reaction volume. We selected optimized Accellerase XC mix for the scale up validation as it predicted the same enzyme components (Accellerase XC, FAE 1a and AXE 16A) (Figures 3c and 4c) for enhanced glucose as well as xylose from barley straw. Superior performance of optimized Accellerase XC over Accellerase XC was confirmed in the scale up through the observation of increased glucose and xylose release (Figure 7a, b). A direct relationship was observed between xylan conversion (fraction of available xylan converted) and glucose conversion (fraction of available glucan conversion) for Accellerase XC as well as for optimized Accellerase XC (Figure 8a, and Additional file 5). However, the correlation between xylan and glucan digestion in optimized Accellerase XC (Figure 8b) was higher than conventional Accellerase XC, suggesting improved glucan conversion as a result of enhanced xylan saccharification.. Since FAE 1A and AXE 16A were important components of optimized Accellerase XC, we further analysed the release of acetate. Accellerase XC exhibited a weak correlation between xylose and acetate release, while there was a strong correlation between xylose and acetate release with optimized Accellerase XC. The synergetic relationship between Accellerase XC with FAE 1a and AXE 16A was evident by the direct relationship between xylose release and acetate release during incubation (Figure 8c, d). These results suggest that Accellerase XC lacked sufficient esterase activity to effectively, hydrolyze AP pretreated barley straw, a limitation that was overcome in part by the addition of complementary esterase activity.

## Conclusions

Fungal esterases (FAE1a and AXE16A_ASPNG) improved the activity of both commercial enzymes in this study. Our results demonstrate that the breakdown of hemicellulose facilitates the hydrolysis of barley cellulose. Addition of endoglucanase (EGL7A_THITE) enhanced the activity of commercial enzymes against both alfalfa hay and barley straw. Use of enzymes from thermophilic or anaerobic fungi to formulate "intelligent" enzyme cocktails could enhance the efficacy of existing commercial enzyme preparations. Characterization of enzymes that act synergistically with enzymes produced by rumen microorganisms could also lead to an improvement in the utilization of low quality forages by ruminants.

## Methods

### Enzymes

Two acetyl xylan esterases, AXE16A_ASPNG and AXE16B_ASPNG; a polygalacturonase (PGA28A_ASPNG), a α-arabinofuranosidase (ABF54B_ASPNG); an endoglucanase GH7 (EGL7A_THITE) and an endoxylanase (XYN11A_THITE) were produced as recombinant proteins in *A. niger*. Recombinant feruloyl esterase (FAE 1a) was cloned into the pET30a expression vector and overexpressed in *Escherichia coli* BL21 (DE3). β-glucosidase (E-BGLUC) and β-xylosidase (E-BXSRB) were procured from Megazyme International (Bray, Ireland). Mixed rumen enzymes were prepared from rumen fluid collected from two rumen cannulated cows fed a mixed alfalfa hay (30%), barley silage (50%) and barley grain (20%; DM basis) diet. Rumen contents were collected 2 h after feeding from the reticulum, ventral, caudal and dorsal-ventral sac of the reticulo-rumen of each cow and thoroughly mixed. Contents were strained through 4 layers of cheesecloth and the collected fluid was centrifuged at 38,300 × *g* for 15 min. The supernatant (500 mL) was lyophilized and reconstituted in 50 mM sodium citrate (pH 5.0, containing 5 μg/mL tetracycline, 5 μg/mL cycloheximide and 0.02% sodium azide) and used as source of rumen mixed enzymes. Three batches of rumen mixed enzymes from three different collections of rumen fluid were prepared. The commercial enzyme preparations of Accellerase 1500 and Accellerase XC were obtained from Genencor (Rochester, NY, US). Accellerase 1500 contained endoglucanase (2200–2800 carboxymethycellulose (CMC U) U/g) and β-glucosidase activities (450–775 p-nitrophenyl-β-D-glucopyranoside (pNPG U) U/g) whilst Accellerase XC contained mainly endoglucanase (1000–1400 CMC U/g) and xylanase activity (2500–3800 acid birchwood xylanase units ABXU/g; Genencor, Rochester, NY, US).

### Production of acetyl xylan esterase, polygalacturonase, α-arabinofuranosidase, endoglucanase, endoxylanase in *A. niger*

Nucleotide sequences corresponding to target genes were obtained from Mycocosm genome resource of the Joint Genome Institute ( http://genome.jgi-psf.org/programs/fungi/index.jsf). Additional file 6: Table S1 outlines the origin of the cloned genes and the carbohydrases that they encode. Cloning was accomplished using the Gateway recombination method with Invitrogen enzymes (Life Technologies Inc., Burlington, ON). Forward and reverse primers for cloning possessed at their 3′ end 20–25 nucleotides identical to the N terminal and C-terminal portions of the coding region of the targeted ORF, along with Gateway BP reaction compatible recombination sites [25]. Complementary DNA prepared from poly (A) + RNA was amplified by polymerase chain reaction (PCR). The amplified PCR products were cloned into ANIp7G, a Gateway-compatible vector previously constructed from ANIp7 [26]. Protoplasts of *A. niger* strain N593 *glaA::hisG* were transformed with the target genes as previously described [27]. FAE 1a from *Anaeromyces mucronatus* was cloned, expressed in *E. coli* BL21 and purified as earlier described [28].

### Biochemical characterization of recombinant enzymes

Recombinant proteins produced in *A. niger* were purified at room temperature by anion exchange chromatography using an ÄKTA chromatography system (Amersham Biosciences, Piscataway, NJ). Prior to chromatography, supernatants from *A. niger* cultures were concentrated using a Vivaspin ultrafiltration device (GE Healthcare Life Sciences, Baie d’Urfe, PQ) at a 10 kDa cut-off. The concentrated proteins were repeatedly washed in 20 mM Tris–HCl buffer, pH 8.0, by dilution and ultrafiltration, and the retentate was applied to a MonoQ HR 5/10 anion exchange column equilibrated with the same buffer. Bound proteins were eluted using a linear 0 to 1 M KCl gradient in 20 mM Tris–HCl buffer, pH 8.0. Fractions (1 mL) were collected at a flow rate of 1 mL/min and stored on ice until analyzed for enzyme activity. Purified protein was stored at -80°C for further use. Protein purity was checked by SDS-PAGE as previously outlined [29].

All enzyme reactions were carried out in triplicate. Glycoside hydrolase activity was determined using a reducing sugar assay performed in a 96-well micro plate format with an assay volume of 50 μL. Briefly, for a 50 μL assay, 10 μL of substrate (1%) was added to 30 μL of 50 mM Britton-Robinson buffer (50 mM boric acid, 50 mM acetic acid and 50 mM phosphoric acid) pH 5.0 and the reaction initiated by the addition of the appropriate enzyme diluted in 10 μL of Britton-Robinson buffer [30]. The reaction mixture was immediately incubated at 40˚C for 30 min. Reaction mixtures were placed on ice and 10 μL was withdrawn and mixed with 190 μL of ice-cold BCA reagent (Bicinchoninic acid assay, Sigma-Aldrich, Oakville, ON) and incubated at 80˚C for 40 min for colour development. The resultant mixture (160 μL) was loaded onto a flat bottom-micro plate and the optical density read at 562 nm. The monosaccharide subunits constituting the polysaccharide substrates were used to prepare standard curves. Polysaccharide substrates (all from Sigma-Aldrich unless otherwise specified) were: birchwood xylan (xylanases); carboxymethylcellulose (endoglucanase); and polygalacturonic acid, sodium salt (polygalacturonase). Arabinofuranosidase activity was determined using 4-nitrophenyl-L-arabinofuranoside as a substrate in 50 mM Britton-Robinson buffer, pH 5.0, as described previously [30]. Acetyl-esterase activity was assessed by measuring released acetic acid using a kit purchased from Megazyme.

### Statistical design

Design-Expert® software (Version 8.0; Stat-Ease, Inc., Minneapolis, MN; http://www.statease.com) was used to create the simplex-lattice designs and to analyze responses. All experiments were performed as mixtures at 15 mg protein loadings per g cellulose of the pretreated alfalfa hay or barley straw. The number of mixtures in the simplex-lattice depended on both the number of components in the mixture and the degree of the polynomial. Using an augmented special quadratic design, ten component designs resulted in 66 separate assays. In order to optimize the hydrolysis efficiency of enzyme cocktails the relative abundance of each component was varied using the experimental design outlined in Additional file 7: Table S2. The lower and upper limits of each component were determined with an aim to analyze synergetic interaction among fungal enzymes (AXE16A_ASPNG, AXE16B_ASPNG, PGA28A_ASPNG, ABF54B_ASPNG, EGL7A_THITE, XYN11A_THITE, FAE 1a, E-BGLUC and E-BXSRB) with mixed rumen enzymes or with commercial enzyme preparation to achieve enhanced biomass conversion to fermentable sugar. Therefore, relative abundance of core enzymes (i.e., mixed rumen enzymes/Accellerase 1500/Accellerase XC) was set to vary from 50% to 100%, while upper limit and lower limits for fungal enzymes were set between 50% - 0% in assay mixtures.

### Formulation of enzyme mixtures for effective conversion of alkaline peroxide pre-treated (AP) alfalfa hay and barley straw

#### Alkaline peroxide pre-treatment of alfalfa hay and barley straw

Alfalfa and barley straw were each obtained from a single source of parental material and ground to pass 1.0 mm screen sieve and the resultant particles were pretreated with alkaline peroxide using the procedure described by [8]. Briefly, 50-mL, 1% H_2_O_2_ was adjusted to pH = 11.5 with 5 M NaOH and mixed with 1.0 g of alfalfa hay or barley straw in a 250-mL Erlenmeyer flask. Final concentrations were 1% H_2_O_2_ (300 mM), 0.8% NaOH (200 mM) and 2% (w/v) substrate. The flasks were incubated in a shaking incubator at 24°C for 24 h at 90 rpm. The slurries were neutralized to pH 7 by drop-wise addition of 12-N HCl. Residual H_2_O_2_ was inactivated by addition of 59 μL of catalase (28 mg protein/mL, Sigma–Aldrich). Following inactivation of catalase by heating at 90°C for 15 min, the entire content of the flasks were lyophilized before use in enzymatic assays. Two independent batches of pretreated alfalfa and barley straw were generated.

#### Enzymatic digestion of alkaline peroxide treated alfalfa hay or barley straw

Treated alfalfa hay or barley straw was first suspended at a final concentration of 0.5% in 50 mM sodium citrate (pH = 5.0) containing 5 μg/mL of tetracycline, 5 μg/mL of cycloheximide and 0.02% sodium azide. A total of 200 μL (duplicate) of substrate slurry was dispensed into a mini-eppendorf while the slurry was kept in suspension using a paddle reservoir designed for dispensing pharmaceutical beads on the Biomek FXP (Model VP 756C-1P100, V&P Scientific, Inc., San Diego, CA). Accuracy and precision of the biomass dispensing was tested by drying and weighing a series of dispensed aliquots, each of 0.2 mL.

Respective enzyme volumes for each reaction mixture were calculated according to statistical design (Additional file 7: Table S2) and dispensed into 200 μL of substrate slurry prepared as described above. Final volume was adjusted to 250 μL with 50 mM sodium citrate buffer (pH 5.0, containing 5 μg/mL tetracycline, 5 μg/mL cycloheximide and 0.02% sodium azide), and the reaction mixture was incubated at 50°C for 48 h in an oven on a platform rotating at 10 rpm. The tubes were then centrifuged at 1,500 × *g* for 3 min to separate the solid residue from the digested mixture. The supernatants (100 μL) were transferred into microplate wells (Thermo Fisher Scientific, Rochester, NY) and heated at 90°C for 10 min to inactivate enzymes prior determination of liberated glucose and xylose.

#### Glucose and xylose assays

Free glucose and xylose were determined colorimetrically using enzyme-coupled assays kits supplied by Megazyme (catalog K-GLUC and K-Xylose respectively). Assays were performed in 96-well plates using 12 μL of sample (obtained above) and 194 and 297 μL of assay reagent for glucose and xylose, respectively. Plates containing hydrolysis products and reagent were incubated at 50°C for 20 min and read at 510 nm and 340 nm for glucose and xylose respectively, using a Synergy-HT multi detection micro plate reader (Biotek Instruments, Inc. Winooski, VT). All reactions were replicated once, sampled twice, and assayed twice (n = 8).

For calculating total cellulose content of AP treated alfalfa and barley straw, triplicate alcohol insoluble residues from each feed stock was de-starched using Type-II A *Bacillus α*-amylase (Sigma-Aldrich; ~1000 units/100 mg cell wall alcohol-insoluble residue) in 50 mM sodium phosphate buffer (pH 7.0) at 25°C in a shaking incubator for 48 h. De-starched samples were centrifuged (3660× *g* for 10 min at 25°C) and the pellet was subsequently washed thrice with deionized water followed by centrifugation (3660 × *g* for 10 min at 25°C) and decanting of the water. The resulting pellets were suspended in 500 μl of acetone and evaporated with a stream of air at 36°C until dry. De-starched residue (5 mg) was hydrolyzed with 72% H_2_SO_4_. The released sugars were quantitated by combination of Gas Chromatography/mass spectroscopy (GC/MS) of alditol acetate derivatives [31].

### Data analysis

Absorbance values from the glucose and xylose assays were converted to relative percent yields (relative to controls i.e., commercial enzyme or rumen enzyme mix only) and these values served as responses in the experimental design. For all experiments, ANOVA calculations of *F* value, *P* value, *R*^2^, Adjusted *R*^2^, Predicted *R*^2^ and Adequate Precision were computed by the Design-Expert software as shown in Additional file 8: Table S3 and Additional file 9: Table S4). The *F* value indicated the effects (if any) of the individual components on the model. An *F* value close to 1 implied that the components of the mixture did not interact and hence had little effect on the model. Enzyme synergism was deemed to be present at a *P* value <0.0001. Adjusted *R*^2^ and Predicted *R*^2^ were estimated and if the difference between these values was > 0.2 the model was considered to be over-parameterized and a different order polynomial was tested and/or a backward or stepwise elimination regression was conducted with an ‘alpha out’ value set to 0.1 as suggested by Banerjee et al., 2010 [8]. This statistical approach eliminated all of the terms in the model that were insignificant (*P* > 0.0001) and the model that gave a difference between the Adjusted and Predicted *R*^2^ values <0.2 was used to navigate the design space. Adequate precision estimated the signal-to-noise ratio, with a value >4 indicating adequate model discrimination. Once all the criteria for a robust model were fulfilled, the model was used to determine the enzyme mixtures that resulted in optimal glucose and xylose release. The predicted vs. actual estimates for each design are shown in Additional file 10.

## Competing interests

The authors declare that they have no competing interests.

## Authors’ contributions

Conceived and designed the experiment: TM, AT, AB, JP. Performed experiments: AB, DP, GR. Analysed data: AB, TM and YW. Wrote paper: AB, YW, TM, AT. All authors read and approved the final manuscript.

## Supplementary Material

Additional file 1 Optimization of enzyme mixtures for relative glucose yield as a function of synergetic interaction of mixed rumen enzymes with (a), Accellerase 1500 (b), Accellerase XC (c) with recombinant enzymes for hydrolysis of alkaline peroxide pre-treated alfalfa. Click here for file

Additional file 2 Optimization of enzyme mixtures for relative xylose yield as a function of synergetic interaction of rumen enzymes mix (a), Accellerase 1500 (b), Accellerase XC (c) with recombinant enzymes for hydrolysis of alkaline peroxide pre-treated alfalfa. Click here for file

Additional file 3 Optimization of enzyme mixtures for relative glucose yield as a function of synergetic interaction of rumen enzymes mix (a), Accellerase 1500 (b), Accellerase XC (c) with recombinant enzymes for hydrolysis of alkaline peroxide pre-treated barley straw. Click here for file

Additional file 4 Optimization of enzyme mixtures for relative xylose yield as a function of synergetic interaction of rumen enzymes mix (a), Accellerase 1500 (b), Accellerase XC (c) with recombinant fungal enzymes for hydrolysis of alkaline peroxide pre-treated barley straw. Click here for file

Additional file 5 **Average of glucose release plotted against average of xylose release after 48 h of incubation of barley straw (a) and alfalfa hay (b) with optimized enzyme mix (i.e. average of optimized Accellerase 1500 and optimized Accellerase XC).** Optimized Accellerase 1500 and optimized Accellerase XC composition was identical to those which generated Figures 1b, 3b and Figures 1c, 3c), respectively. Enzymes were included at 15 mg/g glucan. Click here for file

Additional file 6: Table S1 Gene source, activity and characteristics of select carbohydrases examined in the current study. Click here for file

Additional file 7: Table S2 Experimental design for ten component experiment. Click here for file

Additional file 8: Table S3 ANOVA calculations of *F*-value, *P*-value, *R*^2^, Adjusted *R*^2^, Predicted *R*^2^, and Adequate Precision as calculated by the Design-Expert software for *Glucose Released.* Click here for file

Additional file 9: Table S4 ANOVA calculations of *F*-value, *P*-value, *R*^2^, Adjusted *R*^2^, Predicted *R*^2^, and Adequate Precision as calculated by the Design-Expert software for *Xylose Released*. Click here for file

Additional file 10 Predicted vs. Actual plots for design of experiment for glucose yield from pretreated alfalfa and barley (Row 1 & 3 respectively), xylose yield from pretreated alfalfa and barley (lane 2 & 4 respectively) using rumen enzyme mix, Accellerase 1500 and Accellerase XC. Click here for file
