# Supplementary material for: Formulation of enzyme blends to maximize the hydrolysis of alkaline peroxide pretreated alfalfa hay and barley straw by rumen enzymes and commercial cellulases
Source: BMC Biotechnol. 2014 Apr 26;14:31. doi: 10.1186/1472-6750-14-31 (PMC4022426; doi:10.1186/1472-6750-14-31)
Supplement: Additional file 1 — Optimization of enzyme mixtures for relative glucose yield as a function of synergetic interaction of mixed rumen enzymes with (a), Accellerase 1500 (b), Accellerase XC (c) with recombinant enzymes for hydrolysis of alkaline peroxide pre-treated alfalfa. [file 1472-6750-14-31-S1.docx]

Prediction: 429.7

SE Mean: 6.12

SE Pred: 8.69

Prediction: 160.7

SE Mean: 0.966

SE Pred: 1.42

Prediction: 708.9

SE Mean: 132.8

SE Pred: 136.5

Additional file 2 Figure 2: Optimization of enzyme mixtures for relative xylose yield as a function of synergetic interaction of rumen enzymes mix (a), Accellerase 1500(b), Accellerase XC (c) with recombinant enzymes for hydrolysis of alkaline peroxide pre-treated alfalfa.

Badhan et al
