# Supplementary material for: Formulation of enzyme blends to maximize the hydrolysis of alkaline peroxide pretreated alfalfa hay and barley straw by rumen enzymes and commercial cellulases
Source: BMC Biotechnol. 2014 Apr 26;14:31. doi: 10.1186/1472-6750-14-31 (PMC4022426; doi:10.1186/1472-6750-14-31)
Supplement: Additional file 2 — Optimization of enzyme mixtures for relative xylose yield as a function of synergetic interaction of rumen enzymes mix (a), Accellerase 1500 (b), Accellerase XC (c) with recombinant enzymes for hydrolysis of alkaline peroxide pre-treated alfalfa. [file 1472-6750-14-31-S2.docx]

Prediction: 123.4

SE Mean: 0.25

SE Pred: 0.40

Prediction: 186

SE Mean: 0.88

SE Pred: 1.25

Prediction: 160.4

SE Mean: 0.95

SE Pred: 1.53

Additional file 3. Figure 3: Optimization of enzyme mixtures for relative glucose yield as a function of synergetic interaction of rumen enzymes mix (a), Accellerase 1500(b), Accellerase XC (c) with recombinant enzymes for hydrolysis of alkaline peroxide pre-treated barley straw.

Badhan et al
