# Supplementary material for: Formulation of enzyme blends to maximize the hydrolysis of alkaline peroxide pretreated alfalfa hay and barley straw by rumen enzymes and commercial cellulases
Source: BMC Biotechnol. 2014 Apr 26;14:31. doi: 10.1186/1472-6750-14-31 (PMC4022426; doi:10.1186/1472-6750-14-31)
Supplement: Additional file 3 — Optimization of enzyme mixtures for relative glucose yield as a function of synergetic interaction of rumen enzymes mix (a), Accellerase 1500 (b), Accellerase XC (c) with recombinant enzymes for hydrolysis of alkaline peroxide pre-treated barley straw. [file 1472-6750-14-31-S3.docx]

Prediction: 141

SE Mean: 3.21

SE Pred: 3.41

Prediction: 145.2

SE Mean: 1.27

SE Pred: 2.08

Prediction: 505.3

SE Mean: 110.2

SE Pred: 112.9

Additional file 4. Figure 4: Optimization of enzyme mixtures for relative xylose yield as a function of synergetic interaction of rumen enzymes mix (a), Accellerase 1500(b), Accellerase XC (c) with recombinant fungal enzymes for hydrolysis of alkaline peroxide pre-treated barley straw.

Badhan et al
