# Supplementary material for: Formulation of enzyme blends to maximize the hydrolysis of alkaline peroxide pretreated alfalfa hay and barley straw by rumen enzymes and commercial cellulases
Source: BMC Biotechnol. 2014 Apr 26;14:31. doi: 10.1186/1472-6750-14-31 (PMC4022426; doi:10.1186/1472-6750-14-31)
Supplement: Additional file 4 — Optimization of enzyme mixtures for relative xylose yield as a function of synergetic interaction of rumen enzymes mix (a), Accellerase 1500 (b), Accellerase XC (c) with recombinant fungal enzymes for hydrolysis of alkaline peroxide pre-treated barley straw. [file 1472-6750-14-31-S4.docx]

Additional Figure 5:
