# Supplementary material for: Formulation of enzyme blends to maximize the hydrolysis of alkaline peroxide pretreated alfalfa hay and barley straw by rumen enzymes and commercial cellulases
Source: BMC Biotechnol. 2014 Apr 26;14:31. doi: 10.1186/1472-6750-14-31 (PMC4022426; doi:10.1186/1472-6750-14-31)
Supplement: Additional file 5 — Average of glucose release plotted against average of xylose release after 48 h of incubation of barley straw (a) and alfalfa hay (b) with optimized enzyme mix (i.e. average of optimized Accellerase 1500 and optimized Accellerase XC). Optimized Accellerase 1500 and optimized Accellerase XC composition was identical to those which generated Figures 1b, 3b and Figures 1c, 3c), respectively. Enzymes were included at 15 mg/g glucan. [file 1472-6750-14-31-S5.docx]

Additional file 6: Table S1: Gene source, activity and characteristics of select carbohydrases examined in the current study

| **Clone ID** | **Entry name** | **Enzyme activity** | **CAZy family** | **Organism** | **mycoCLAP entry name** | **JGI ID** | **UniProt ID** | **pH optimum** | **temp opt** |
| --- | --- | --- | --- | --- | --- | --- | --- | --- | --- |
| Anig_Anig200605C | ABF54B_ASPNG | alpha-N-arabinofuranosidase | GH54 | Aspergillus niger N400 | ABF54B_ASPNG | 200605^a^ | P42255 | 4.5 | 50 |
| Anig_Anig214598C | PGA28A_ASPNG | endopolygalacturonase | GH28 | Aspergillus niger N400 | PGA28A_ASPNG | 214598^a^ | Q9P4W4 | 4 | 60 |
| Asn194096 | AXE16B_ASPNG | acetylesterase | CE16 | Aspergillus niger N400 | TBS | 194096^a^ | G3Y7Z1 | 5 | 60 |
| Asn7870 | AXE16A_ASPNG | acetylesterase | CE16 | Aspergillus niger N400 | TBS | 54865^a^ | G3Y497 | 6 | 55 |
| Anig_TterXXXX28G | XYN11A_THITE | endo-1,4-beta-xylanase | GH11 | Thielavia terrestris | TBS | 154456^b^ | G2QV82 | 5.5 | 45 |
| Anig_TterXXXX7G | EGL7A_THITE | endoglucanase | GH7 | Thielavia terrestris | TBS | 54138^b^ | G2QZA7 | 6 | 55 |

^a^The ID number corresponds to the protein ID of *Aspergillus niger* v3.0 annotation, <http://genome.jgi-psf.org/Aspni5>; ^b^the ID number corresponds to the protein ID of *Thielavia terrestris* v3.0 annotation, <http://genome.jgi-psf.org/Thite2>.

Badhan et al
