# Supplementary material for: Formulation of enzyme blends to maximize the hydrolysis of alkaline peroxide pretreated alfalfa hay and barley straw by rumen enzymes and commercial cellulases
Source: BMC Biotechnol. 2014 Apr 26;14:31. doi: 10.1186/1472-6750-14-31 (PMC4022426; doi:10.1186/1472-6750-14-31)
Supplement: Additional file 6: Table S1 — Gene source, activity and characteristics of select carbohydrases examined in the current study. [file 1472-6750-14-31-S6.docx]

Additional file 7: Table S2: Experimental design for ten component experiment

|  |  | **Component**  **1** | **Component**  **2** | **Component**  **3** | **Component**  **4** | **Component**  **5** | **Component**  **6** | **Component**  **7** | **Component**  **8** | **Component 9** | **Component 10** |
| --- | --- | --- | --- | --- | --- | --- | --- | --- | --- | --- | --- |
| **Std** | **Run** | **A:Coreenzyme (RF/AC1500/AC-XC)** | **B:FAE 1a** | **C: AXE16A_ASPNG** | **D: AXE16B_ASPNG** | **E: EGL7A_THITE** | **F: XYN11A_THITE** | **G: ABF54B_ASPNG** | **H: PGA28A_ASPNG** | **J:E-BGLU** | **K:E-BXSRB** |
| 65 | 1 | 0.525 | 0.025 | 0.025 | 0.025 | 0.025 | 0.025 | 0.025 | 0.025 | 0.025 | 0.275 |
| 23 | 2 | 0.5 | 0.25 | 0 | 0 | 0 | 0.25 | 0 | 0 | 0 | 0 |
| 64 | 3 | 0.525 | 0.025 | 0.025 | 0.025 | 0.025 | 0.025 | 0.025 | 0.025 | 0.275 | 0.025 |
| 21 | 4 | 0.5 | 0.25 | 0 | 0.25 | 0 | 0 | 0 | 0 | 0 | 0 |
| 52 | 5 | 0.5 | 0 | 0 | 0 | 0 | 0 | 0.25 | 0 | 0 | 0.25 |
| 9 | 6 | 0.5 | 0 | 0 | 0 | 0 | 0 | 0 | 0 | 0.5 | 0 |
| 2 | 7 | 0.5 | 0.5 | 0 | 0 | 0 | 0 | 0 | 0 | 0 | 0 |
| 12 | 8 | 0.75 | 0 | 0.25 | 0 | 0 | 0 | 0 | 0 | 0 | 0 |
| 32 | 9 | 0.5 | 0 | 0.25 | 0 | 0 | 0 | 0 | 0.25 | 0 | 0 |
| 47 | 10 | 0.5 | 0 | 0 | 0 | 0 | 0.25 | 0 | 0.25 | 0 | 0 |
| 51 | 11 | 0.5 | 0 | 0 | 0 | 0 | 0 | 0.25 | 0 | 0.25 | 0 |
| 28 | 12 | 0.5 | 0 | 0.25 | 0.25 | 0 | 0 | 0 | 0 | 0 | 0 |
| 50 | 13 | 0.5 | 0 | 0 | 0 | 0 | 0 | 0.25 | 0.25 | 0 | 0 |
| 41 | 14 | 0.5 | 0 | 0 | 0 | 0.25 | 0.25 | 0 | 0 | 0 | 0 |
| 38 | 15 | 0.5 | 0 | 0 | 0.25 | 0 | 0 | 0 | 0.25 | 0 | 0 |
| 60 | 16 | 0.525 | 0.025 | 0.025 | 0.025 | 0.275 | 0.025 | 0.025 | 0.025 | 0.025 | 0.025 |
| 40 | 17 | 0.5 | 0 | 0 | 0.25 | 0 | 0 | 0 | 0 | 0 | 0.25 |
| 15 | 18 | 0.75 | 0 | 0 | 0 | 0 | 0.25 | 0 | 0 | 0 | 0 |
| 26 | 19 | 0.5 | 0.25 | 0 | 0 | 0 | 0 | 0 | 0 | 0.25 | 0 |
| 29 | 20 | 0.5 | 0 | 0.25 | 0 | 0.25 | 0 | 0 | 0 | 0 | 0 |
| 1 | 21 | 1 | 0 | 0 | 0 | 0 | 0 | 0 | 0 | 0 | 0 |
| 30 | 22 | 0.5 | 0 | 0.25 | 0 | 0 | 0.25 | 0 | 0 | 0 | 0 |
| 39 | 23 | 0.5 | 0 | 0 | 0.25 | 0 | 0 | 0 | 0 | 0.25 | 0 |
| 13 | 24 | 0.75 | 0 | 0 | 0.25 | 0 | 0 | 0 | 0 | 0 | 0 |
| 42 | 25 | 0.5 | 0 | 0 | 0 | 0.25 | 0 | 0.25 | 0 | 0 | 0 |
| 56 | 26 | 0.775 | 0.025 | 0.025 | 0.025 | 0.025 | 0.025 | 0.025 | 0.025 | 0.025 | 0.025 |
| 4 | 27 | 0.5 | 0 | 0 | 0.5 | 0 | 0 | 0 | 0 | 0 | 0 |
| 14 | 28 | 0.75 | 0 | 0 | 0 | 0.25 | 0 | 0 | 0 | 0 | 0 |
| 18 | 29 | 0.75 | 0 | 0 | 0 | 0 | 0 | 0 | 0 | 0.25 | 0 |
| 36 | 30 | 0.5 | 0 | 0 | 0.25 | 0 | 0.25 | 0 | 0 | 0 | 0 |
| 7 | 31 | 0.5 | 0 | 0 | 0 | 0 | 0 | 0.5 | 0 | 0 | 0 |
| 61 | 32 | 0.525 | 0.025 | 0.025 | 0.025 | 0.025 | 0.275 | 0.025 | 0.025 | 0.025 | 0.025 |
| 66 | 33 | 0.55 | 0.05 | 0.05 | 0.05 | 0.05 | 0.05 | 0.05 | 0.05 | 0.05 | 0.05 |
| 25 | 34 | 0.5 | 0.25 | 0 | 0 | 0 | 0 | 0 | 0.25 | 0 | 0 |
| 45 | 35 | 0.5 | 0 | 0 | 0 | 0.25 | 0 | 0 | 0 | 0 | 0.25 |
| 46 | 36 | 0.5 | 0 | 0 | 0 | 0 | 0.25 | 0.25 | 0 | 0 | 0 |
| 17 | 37 | 0.75 | 0 | 0 | 0 | 0 | 0 | 0 | 0.25 | 0 | 0 |
| 5 | 38 | 0.5 | 0 | 0 | 0 | 0.5 | 0 | 0 | 0 | 0 | 0 |
| 54 | 39 | 0.5 | 0 | 0 | 0 | 0 | 0 | 0 | 0.25 | 0 | 0.25 |
| 37 | 40 | 0.5 | 0 | 0 | 0.25 | 0 | 0 | 0.25 | 0 | 0 | 0 |
| 33 | 41 | 0.5 | 0 | 0.25 | 0 | 0 | 0 | 0 | 0 | 0.25 | 0 |
| 55 | 42 | 0.5 | 0 | 0 | 0 | 0 | 0 | 0 | 0 | 0.25 | 0.25 |
| 48 | 43 | 0.5 | 0 | 0 | 0 | 0 | 0.25 | 0 | 0 | 0.25 | 0 |
| 49 | 44 | 0.5 | 0 | 0 | 0 | 0 | 0.25 | 0 | 0 | 0 | 0.25 |
| 62 | 45 | 0.525 | 0.025 | 0.025 | 0.025 | 0.025 | 0.025 | 0.275 | 0.025 | 0.025 | 0.025 |
| 20 | 46 | 0.5 | 0.25 | 0.25 | 0 | 0 | 0 | 0 | 0 | 0 | 0 |
| 31 | 47 | 0.5 | 0 | 0.25 | 0 | 0 | 0 | 0.25 | 0 | 0 | 0 |
| 3 | 48 | 0.5 | 0 | 0.5 | 0 | 0 | 0 | 0 | 0 | 0 | 0 |
| 24 | 49 | 0.5 | 0.25 | 0 | 0 | 0 | 0 | 0.25 | 0 | 0 | 0 |
| 34 | 50 | 0.5 | 0 | 0.25 | 0 | 0 | 0 | 0 | 0 | 0 | 0.25 |
| 27 | 51 | 0.5 | 0.25 | 0 | 0 | 0 | 0 | 0 | 0 | 0 | 0.25 |
| 11 | 52 | 0.75 | 0.25 | 0 | 0 | 0 | 0 | 0 | 0 | 0 | 0 |
| 8 | 53 | 0.5 | 0 | 0 | 0 | 0 | 0 | 0 | 0.5 | 0 | 0 |
| 53 | 54 | 0.5 | 0 | 0 | 0 | 0 | 0 | 0 | 0.25 | 0.25 | 0 |
| 58 | 55 | 0.525 | 0.025 | 0.275 | 0.025 | 0.025 | 0.025 | 0.025 | 0.025 | 0.025 | 0.025 |
| 22 | 56 | 0.5 | 0.25 | 0 | 0 | 0.25 | 0 | 0 | 0 | 0 | 0 |
| 19 | 57 | 0.75 | 0 | 0 | 0 | 0 | 0 | 0 | 0 | 0 | 0.25 |
| 63 | 58 | 0.525 | 0.025 | 0.025 | 0.025 | 0.025 | 0.025 | 0.025 | 0.275 | 0.025 | 0.025 |
| 43 | 59 | 0.5 | 0 | 0 | 0 | 0.25 | 0 | 0 | 0.25 | 0 | 0 |
| 16 | 60 | 0.75 | 0 | 0 | 0 | 0 | 0 | 0.25 | 0 | 0 | 0 |
| 57 | 61 | 0.525 | 0.275 | 0.025 | 0.025 | 0.025 | 0.025 | 0.025 | 0.025 | 0.025 | 0.025 |
| 6 | 62 | 0.5 | 0 | 0 | 0 | 0 | 0.5 | 0 | 0 | 0 | 0 |
| 35 | 63 | 0.5 | 0 | 0 | 0.25 | 0.25 | 0 | 0 | 0 | 0 | 0 |
| 44 | 64 | 0.5 | 0 | 0 | 0 | 0.25 | 0 | 0 | 0 | 0.25 | 0 |
| 10 | 65 | 0.5 | 0 | 0 | 0 | 0 | 0 | 0 | 0 | 0 | 0.5 |
| 59 | 66 | 0.525 | 0.025 | 0.025 | 0.275 | 0.025 | 0.025 | 0.025 | 0.025 | 0.025 | 0.025 |

Badhan et al
