# Supplementary material for: Formulation of enzyme blends to maximize the hydrolysis of alkaline peroxide pretreated alfalfa hay and barley straw by rumen enzymes and commercial cellulases
Source: BMC Biotechnol. 2014 Apr 26;14:31. doi: 10.1186/1472-6750-14-31 (PMC4022426; doi:10.1186/1472-6750-14-31)
Supplement: Additional file 7: Table S2 — Experimental design for ten component experiment. [file 1472-6750-14-31-S7.docx]

Additional file 8: Table S3: ANOVA calculations of *F*-value, *P*-value, *R*^2^, Adjusted *R*^2^, Predicted *R*^2^, and Adequate Precision as calculated by the Design-Expert software for *Glucose Released*

| **Feed-stock** | **Enzyme source** | **F-value** | **P-value** | **R-square** | **Adjusted R-square** | **Predicted R-square** | **Difference between Adj and Pred R-Square** | **Adequate Precision** |
| --- | --- | --- | --- | --- | --- | --- | --- | --- |
| Alfalfa | Rumen Enzyme mix +fungal enzymes | 158.1 | <0.0001 | 0.99 | 0.98 | 0.91 | 0.07 | 54.2 |
|  | Accellerase 1500+fungal enzymes | 136.8 | <0.0001 | 0.99 | 0.98 | 0.80 | 0.18 | 62.07 |
|  | Accellerase XC + fungal enzymes | 315.91 | <0.0001 | 0.99 | 0.99 | 0.83 | 0.16 | 111.56 |
| Barley | Rumen Enzyme mix + fungal enzymes | 401.7 | <0.0001 | 0.99 | 0.99 | 0.81 | 0.18 | 97.16 |
|  | Accellerase 1500 + fungal enzymes | 958.1 | <0.0001 | 0.99 | 0.99 | 0.88 | 0.11 | 193.0 |
|  | Accellerase XC + fungal enzymes | 327.1 | <0.0001 | 0.99 | 0.99 | 0.86 | 0.13 | 143.5 |

Badhan et al
