# Supplementary material for: Formulation of enzyme blends to maximize the hydrolysis of alkaline peroxide pretreated alfalfa hay and barley straw by rumen enzymes and commercial cellulases
Source: BMC Biotechnol. 2014 Apr 26;14:31. doi: 10.1186/1472-6750-14-31 (PMC4022426; doi:10.1186/1472-6750-14-31)
Supplement: Additional file 8: Table S3 — ANOVA calculations of F-value, P-value, R2, Adjusted R2, Predicted R2, and Adequate Precision as calculated by the Design-Expert software for Glucose Released. [file 1472-6750-14-31-S8.docx]

Additional file 9: Table S4: ANOVA calculations of *F*-value, *P*-value, *R*^2^, Adjusted *R*^2^, Predicted *R*^2^, and Adequate Precision as calculated by the Design-Expert software for *Xylose* *Released*

| **Feed- Stock** | **Enzyme source** | **F-value** | **P-value** | **R-square** | **Adjusted R-square** | **Predicted R-square** | **Difference between Adj and Pred R-Square** | **Adequate Precision** |
| --- | --- | --- | --- | --- | --- | --- | --- | --- |
| Alfalfa | Rumen Enzyme mix + fungal enzymes | 33.86 | <0.0001 | 0.94 | 0.91 | 0.79 | 0.12 | 29.78 |
|  | Accellerase + fungal enzymes 1500 | 107.95 | <0.0001 | 0.99 | 0.98 | 0.79 | 0.01 | 79.06 |
|  | Accellerase XC + fungal enzymes | 643.50 | <0.0001 | 0.99 | 0.99 | 0.83 | 0.1650 ucose Releasedpre r-squ | 111.65 |
| Barley | Rumen Enzyme mix + fungal enzymes | 28.65 | <0.0001 | 0.94 | 0.90 | 0.76 | 0.14 | 21.85 |
|  | Accellerase 1500 + fungal enzymes | 173.85 | <0.0001 | 0.99 | 0.99 | 0.82 | 0.17 | 70.97 |
|  | Accellerase XC + fungal enzymes | 94.2 | <0.0001 | 0.99 | 0.98 | 0.81 | 0.17 | 70.53 |

Badhan et al
