# Supplementary material for: Formulation of enzyme blends to maximize the hydrolysis of alkaline peroxide pretreated alfalfa hay and barley straw by rumen enzymes and commercial cellulases
Source: BMC Biotechnol. 2014 Apr 26;14:31. doi: 10.1186/1472-6750-14-31 (PMC4022426; doi:10.1186/1472-6750-14-31)
Supplement: Additional file 9: Table S4 — ANOVA calculations of F-value, P-value, R2, Adjusted R2, Predicted R2, and Adequate Precision as calculated by the Design-Expert software for Xylose Released. [file 1472-6750-14-31-S9.docx]

Rumen enzyme mix↓ Acell 1500↓ Acell XC↓

Row 1

Row2

Row 3

Row 4

Additional file 10

Figure 5 Predicted vs. Actual plots for design of experiment for glucose yield from pretreated alfalfa and barley (Row 1 &3 respectively), xylose yield from pretreated alfalfa and barley (lane 2 &4 respectively) using rumen enzyme mix, Accellerase 1500 and Accellerase XC.

Badhan et al
